# Automated Atrial Fibrillation Detection with ECG

**DOI:** 10.3390/bioengineering9100523

**Published:** 2022-10-05

**Authors:** Ting-Ruen Wei, Senbao Lu, Yuling Yan

**Affiliations:** 1 School of Engineering, Santa Clara University, Santa Clara, CA 95053, USA; 2 Worcester Polytechnic Institute, Worcester, MA 01609, USA

**Keywords:** ECG, A-fib detection, transfer learning

## Abstract

An electrocardiography system records electrical activities of the heart, and it is used to assist doctors in the diagnosis of cardiac arrhythmia such as atrial fibrillation. This study presents a fast, automated deep-learning algorithm that predicts atrial fibrillation with excellent performance (F-1 score 88.2% and accuracy 97.3%). Our approach involves the pre-processing of ECG signals, followed by an alternative representation of the signals using a spectrogram, which is then fed to a fine-tuned EfficientNet B0, a pre-trained convolution neural network model, for the classification task. Using the transfer learning approach and with fine-tuning of the EfficientNet, we optimize the model to achieve highly efficient and effective classification of the atrial fibrillation.

## 1. Introduction

Electrocardiography (ECG) is a noninvasive and effective diagnostic tool; the ECG signal-based interpretation of normal cardiac rhythm and arrhythmia has become common place in healthcare since 1960s and with great progress having been made in the past four decades.

The remarkable advances in computing power and recent breakthroughs in deep-learning technologies have transformed the healthcare industry and medicine. In particular, with the emergence of many top-performing convolutional neural network (CNN) models trained on ImageNet, a benchmark dataset for computer vision, we can apply the learned representations to our tasks via transfer learning, which will expedite the training process and improve the performance of the model [1]. The breakthrough performance of CNN models in image-based analyses have made huge impact in the medical diagnosis arena. Extensive applications have been found in the early detection of a variety of diseases, to list a few, skin cancer, lung nodule, breast tumor, and brain aneurysm.

On the other hand, remarkable progress has been made in the areas of signal processing, especially natural language processing and speech recognition, with the invention of recurrent neural networks (RNN) and long short-term memory (LSTM) networks [2]. LSTM was the first RNN to win the pattern recognition contests [3]. Later, it started to revolutionize speech recognition, outperforming traditional models in several speech recognition applications. Researchers have also attempted to apply LSTM networks to the classification of ECG patterns. For example, Singh et al. utilized RNN and LSTM models to separate regular from irregular beats of the heart and achieved 88.1% in accuracy, 92.4% in sensitivity, and 85.7% in specificity [4], and Faust et al. designed a bidirectional LSTM model to detect atrial fibrillation (A-fib) based on RR intervals, resulting in an accuracy of 98.51%, recall of 98.32%, and specificity of 98.67% in cross validation [5]. 

A-fib is a supraventricular tachyarrhythmia with uncoordinated atrial activation and consequently ineffective atrial contraction [6] and is the most common of the serious cardiac rhythm disturbances [7]. A-fib has a significant impact on longevity, increasing all-cause and cardiovascular mortality rates [8,9] and can lead to blood clots, stroke [10], heart failure and other heart-related complications. It is estimated that 2.7 to 6.1 million people in the United States have A-fib with an expectation to increase, where 2% of people under the age of 65 and 9% of people above age of 65 have A-fib [6]. A-fib costs the United States about USD 6 billion each year and USD 8705 more for each A-fib patient than other patients without A-fib [6,11].

With more and more biological data and health records generated, there is a rising need for effective analyses of these data to find hidden patterns that could potentially provide more insight in human diseases and help design more diagnostic tools and treatment options for complex diseases. A-fib is among one such disease. Over 10% of patients with hypertension but no A-fib history were detected as having atrial tachyarrhythmias, which were associated with an increased risk of clinical atrial fibrillation [12]. Early detection of A-fib could reduce the medical cost and even save lives.

In the current medical practice, an A-fib is determined by a medical doctor from a 12-lead ECG graph with the patterns of irregular R-R intervals (when atrioventricular (AV) conduction is present), absence of distinct repeating P waves, and irregular atrial activity [6]. Non-ECG-based measurements such as blood pressure is also viable, as Verberk et al. showed a recall of 98% and a specificity of 92% for A-fib detection using a blood pressure monitor [13]. However, a lot of work has also been performed for the automatic detection of A-fib from ECG signals without the presence of doctors. Rincon et al. achieved a recall of 96% and a specificity of 93% using a real-time detection method from a wearable wireless sensor platform [14]; Hong et al. achieved an F1 score of 84% using expert features and Deep Neural Networks [15]; and Ribeiro at el. built a unidimensional CNN similar to a residual network that achieved over 80% on F1 scores for multiple classes [16]. A review on current deep-learning ECG models for arrhythmia classification by Ebrahimi, Zahra et al. [17] compiled works from other researchers, their methods, and performances. Specifically, using CNN and the same dataset as the one used in this study, Plesinger et al. achieved an overall F1 score of 81% using statistical descriptions of RR intervals to train a custom 2-layer neural network (NN) and a bagged tree ensemble (BT) [18], and Kamaleswaran et al. reported an average F1 score of 83% and an accuracy of 85.99% using a 13-layer one-dimensional CNN [19]. On the other hand, Andreotti et al. obtained an average F1 score of 83% on a hidden test set by training ResNet on standardized ECG signals supplemented by other PhysioNet databases [20], Fan et al. achieved high accuracy and F1 score (96.99% and 88.78% on 5-s recordings and 98.13% and 92.76% on 20-s recordings), referencing the VGGNet approach [21], and Xiong et al. reached an F1 score of 82% with a unidimensional 16-layer network [22]. Using the same dataset, Maknickas and Maknickas implemented an LSTM network to classify ECG signals with specific QRS features and reported an average F1 score of 78% [23]. Aside from Fan et al., who tackled the problem as a binary classification between A-fib and normal, the others worked on a four-class classification and reported the averaged F1 score for the first three classes (A-fib, normal, other) as the PhysioNet challenge suggested. All of these methods achieved reasonable classifier performance, but deeper and higher-level features from ECG signals were not used in their detection methods. In contrast, our approach uses an alternative time-frequency representation of the raw ECG signals using spectrogram, and our implementation includes the selection of a state-of-the-art model, EfficientNet [24], for the transfer learning and fine-tuning. EfficientNet has a smaller size yet with better accuracy in comparison with previous pre-trained CNN models. In addition, we address the dataset imbalance, an often-encountered crucial issue in medical applications, by introducing class weights to balance the two classes, while most of the above-mentioned work in the literature failed to comment on or address the issue, except for the work of Andreotti et al., who used data augmentation with the inclusion of 2000 A-fib samples from other PhysioNet databases and 2000 simulated noisy samples [20],; similarly, Fan et al. replicated A-fib samples three times in their training set.

Furthermore, we include the Matthews correlation coefficient (MCC) along with other metrics to evaluate our model performance, since MCC is considered the best metric to evaluate classifiers’ performances, especially on imbalanced datasets [25,26].

## 2. Materials and Methods

### 2.1. Data

**Figure 1:**
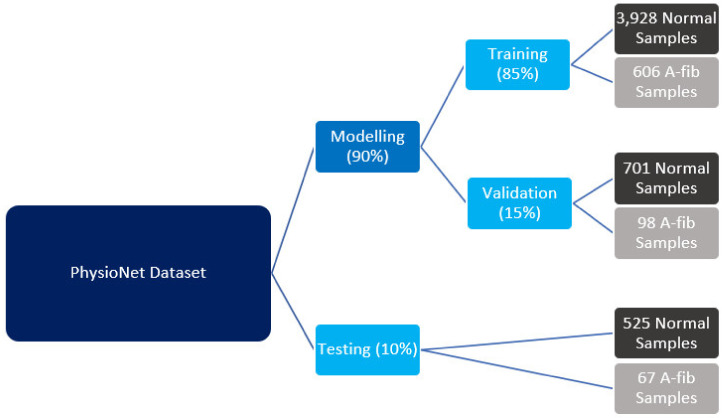
Data split for training, validation, and testing.

In this study, we utilize the dataset from PhysioNet Computing in Cardiology Challenge 2017 [27,28]. The raw dataset contains 8528 data samples, and each sample represents a sequence of time series data sampled at 300 Hz frequency and the samples have various lengths from 9 to 61 s. The summary statistics from the PhysioNet can be found in Table 1 [27,28]. Our study conducts a binary classification that focuses on the 5154 normal and 771 A-fib samples, combining into 5925 samples in total. The normal samples contain no non-A-fib abnormalities [27,28]. There is a mild class imbalance as the majority class represents 87% of the entire dataset. As illustrated in Figure 1, from a total of 5925 samples in this study, we randomly extracted 592 (10%) samples, consisting of 525 normal and 67 A-fib samples, to be our testing set that will be used to evaluate the model performance, leaving 5333 (90%) samples for training and validation of the model. Of the remaining samples, 4534 (85%) and 799 (15%) samples are for training and validation, respectively. The training set consists of 3928 normal and 606 A-fib samples; the validation set consists of 701 normal and 98 A-fib samples. 

**Figure 2:**
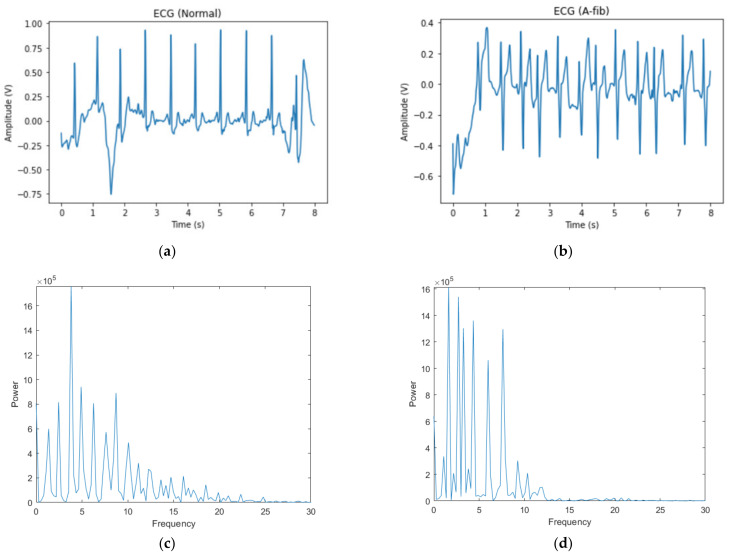
Raw data. (**a**) Normal ECG signal; (**b**) A-fib ECG signal; (**c**) power spectra of the normal ECG signal; (**d**) power spectra of the A-fib ECG signal.

Figure 2 shows a sample ECG signal from each class with their respective power spectra. We observe that there is irregular noise in the raw signals for both classes that can pose a challenge for binary classification. Our first attempt utilized an RNN model on raw ECG signals, and we failed to train a well-performed classifier due to the noises in raw signals, which motivated us to implement a different data representation. In the power spectra for the normal sample, we observe that the energy starts dying out after 20 Hz, and for the A-fib sample, there is high energy before 15 Hz and very little afterwards. The frequency information helps us develop a bandpass filter to clean up the signal, which will be introduced in the Methods section.

### 2.2. Methods

**Figure 3:**
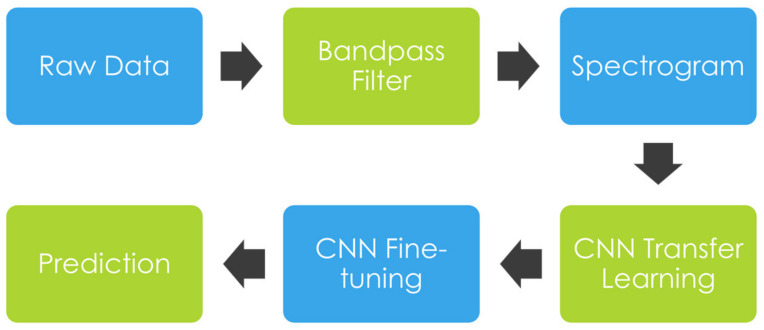
A Diagram of the Process from Pre-processing to Classification.

The raw ECG signals, after the pre-processing step, are converted into image representations using a spectrogram. Subsequently, the image inputs are fed into a CNN model for fine-tuning, as indicated in Figure 3. A detailed description of our methods and implementation is presented below.

#### 2.2.1. Bandpass Filter

We first apply fast Fourier transform (FFT) to our ECG dataset to find a frequency range of interest. From the Fourier spectrum, we observed that there was little energy at frequencies above 20 Hz, so a bandpass filter was applied to the signal to remove the frequencies lower than 0.5 Hz and above 20 Hz. The built-in function bandpass in MATLAB was used to conduct the bandpass filtering of input data. In particular, this is an infinite impulse response (IIR) filter with steepness of 0.95 and stopband attenuation of 60 dB. The filtered signal is then standardized (with zero mean and unit standard deviation).

#### 2.2.2. Spectrogram

**Figure 4:**
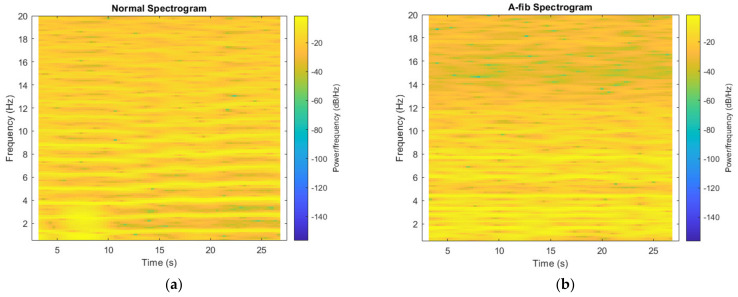
Spectrograms obtained from (**a**) a normal sample; (**b**) an A-fib sample.

Short-term Fourier transform (STFT) is a time-frequency analysis method that transforms the one-dimensional signal into a two-dimensional space, displayed as a spectrogram. A built-in function spectrogram implemented in MATLAB outputs an image, as illustrated in Figure 4. A window size of 2000 and an overlapping width of 1900 samples were used in these analyses, with a sampling rate of 300 Hz. We start the time domain from the 2.5 s mark to remove the noise potentially created in setting up the measurement, and since the median signal length is 30 s, we end the time domain at 27.5 s so the spectrogram widths are more consistent. The color bar was removed from the output graph to yield the image, which, after a grayscale conversion, will be used as the alternative input to the CNN model for learning.

#### 2.2.3. Data Augmentation

**Figure 5:**
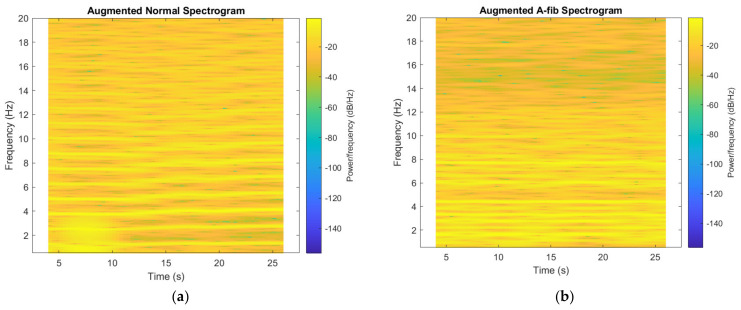
Augmented spectrograms obtained from (**a**) a normal sample; (**b**) an A-fib sample.

As a method for data augmentation, we experimented with creating and adding another set of spectrograms, as shown in Figure 5, to the ones mentioned above, with a different set of parameters: a window size of 2500 and an overlapping width of 2400 samples, which doubles the size of the dataset. Results with and without the augmented dataset are reported in Section 3.2.

#### 2.2.4. CNN Model

**Figure 6:**
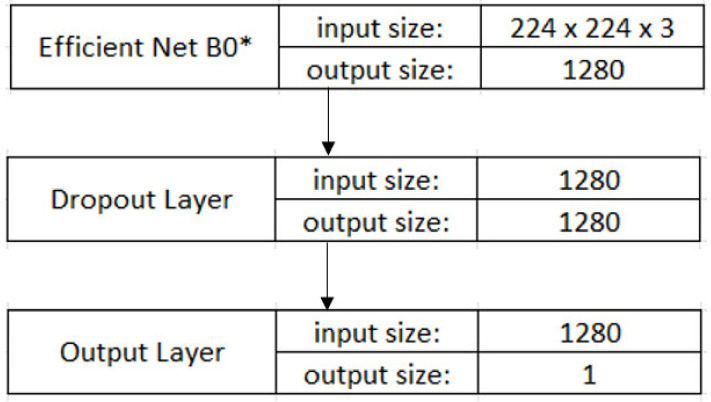
Model architecture (EfficientNet without its last layer). The “*” refers to the “(EfficientNet without its last layer)” in the figure description.

After the data preparation and above-mentioned processing, we fed the spectrograms as input data into a modified EfficientNet B0, a pre-trained CNN model on ImageNet, through the replacement of its output layer with a dropout layer (0.5 dropout rate) and a last layer, which outputs the probability of belonging to the A-fib class. The architecture of the modified EfficientNet model is illustrated in Figure 6. The model contains the weights pretrained on ImageNet and takes input image with a size of 224 × 224 × 3. Our choice of the EfficientNet B0 model is motivated by its high efficiency (smaller size and better accuracy) in comparison with previous state-of-the-art CNN models. For example, the widely used ResNet 50 model by the medical community contains 23,534,592 parameters (in Keras applications), yet it underperforms the smallest EfficientNet model that only takes a total of 5,330,564 parameters [24]. Our previous attempt with GoogLeNet also returned inferior results. Further, with the use of a compound coefficient, EfficientNet models achieved better scaling of the model width, model depth, and the input image resolution [24]. We chose B0, which has the smallest size among the variants of EfficientNet models B0 to B7, considering the small size of our dataset and the fact that the model produced the best results while requiring the least computational resources. To investigate the issue of underfitting, we also experimented with the EfficientNet B1 model which takes an input size of 240 × 240 × 3.

#### 2.2.5. Transfer Learning and Fine-Tuning

With 4629/704 normal/negative to A-fib/positive sample ratio, we encounter a mild dataset imbalance. To address this issue, we use a weight for the minority class that is (4629/704) times larger than that for the majority class in the loss function. 4629 and 704 correspond to the number of normal and A-fib samples, respectively. 

After learning the representations of 1000 classes from ImageNet using the EfficientNet, we intend to transfer some basic knowledge of the representations to our task. Transfer learning can improve the performance by transferring the knowledge learned from a domain to the targeted domain [29]. In particular, with weights pretrained on ImageNet loaded, we froze all layers of the model except the last layer; the model was compiled with the Adam optimizer with a learning rate of 0.001 and was subsequently trained on a binary cross-entropy loss for 53 epochs, determined by our early stopping criteria on validation loss for 20 epochs. The batch size was set to 32.

After the initial training, we then fine-tuned the model by unfreezing the previously frozen layers and training the entire model with the dataset again with the Adam optimizer at a much smaller learning rate of 0.00001 for 72 epochs, determined by taking the maximum validation MCC in 120 epochs, to adjust the overall weights for our task. The overall training time is 91 min for training 4534 images and validation with 799 images on an NVIDIA GTX 1660 Ti GPU.

Instead of utilizing (i) transfer learning and fine-tuning (TL-FT) in a sequential process, we also experimented with (ii) transfer learning as weights initialization (TL-WI) and (iii) using random weights initialization (RWI) with the same EfficientNet B0 model architecture. The TL-WI approach uses the EfficientNet B0 model weights pretrained on ImageNet as starting values of the weights and trains the entire model from there. The RWI approach loads the EfficientNet B0 model with random weights by setting weights = None in Keras. Both experiments involved no freezing of any layer, and as we evaluated the model with the validation set after each training epoch, we saved the model with the maximum validation MCC over 50 epochs as the final model for each experiment. A learning rate of 0.001 was used, and other settings remained the same as those in TL-FT. A comparison of the results is illustrated in Section 3.3.

## 3. Results

A variety of metrics are used to evaluate our model performance and the results are presented below.

### 3.1. Evaluation Metrics

Researchers employ various statistic measures to evaluate the performance of a binary classifier. Some most commonly used measures include accuracy, precision, recall, specificity, and F1 score, which are all computed on the four categories of the confusion matrix: true positives (*TP*), true negatives (*TN*), false positives (*FP*), and false negatives (*FN*).

With A-fib and normal as the positive and negative class, respectively, we first evaluated our model with the following common metrics, as well as the area under the ROC curve (AUC). *TP* and *FP* represent the correctly and incorrectly classified A-fib samples, respectively, and *TN* and *FN* are the same but for normal class. The mathematic formulas for these metrics are shown below.

(1) $$Precision=\frac{TP}{TP+FP}$$

(2) $$Recall=\frac{TP}{TP+FN}$$

(3) $$Accuracy=\frac{TP+TN}{TP+TN+FP+FN}$$

(4) $$Specificity=\frac{TN}{TN+FP}$$

(5) $$F1\;score=\frac{2*TP}{2*TP+FP+FN}$$

While accuracy measures the overall performance on all classes, precision and recall focus on the positive predicted value and the true positive rate, respectively. Recall is a more useful metric when there is class imbalance.

*F1 score* provides the harmonic mean of precision and recall, and the specificity measure evaluates how well the negative class is generalized. The ROC curve, on the other hand, provides a graphic representation that plots the recall against the *FP* rate (1-*specificity*) at multiple threshold values. AUC is obtained from ROC that measures the overall performance of the model.

Although no consensus has been reached on the choice of a single, effective measure, accuracy and F1 score have been the most popular adopted metrics in binary classification tasks. However, these measures can misleadingly show inflated results, especially on imbalanced datasets. The Matthews correlation coefficient (*MCC*), instead, is considered a more reliable measure so that a high score of *MCC* truly reflects a good prediction in terms of all of the four confusion matrix categories (*TP*, *TN*, *FP*, *FN*) and proportional to both the size of positive samples and the size of negative samples in the dataset [25].

The *MCC* measure is defined as follows:

(6) $$MCC=\frac{TP*TN-FP*FN}{\sqrt{\left(TP+FP\right)*\left(TP+FN\right)*\left(TN+FP\right)*\left(TN+FN\right)}}$$

The *MCC* value ranges between −1 and 1; the perfect classification results in an *MCC* of 1.0 and completely incorrect classification results in an *MCC* of −1.0, while a random classification gives a value of 0.

Furthermore, aside from the ROC graphic, we also present the precision–recall (PR) curve, which plots the precision against the recall at multiple threshold values and has been shown to provide more reliable representation of the true classifier performance on imbalanced datasets [26].

### 3.2. Evaluation Results

**Figure 7:**
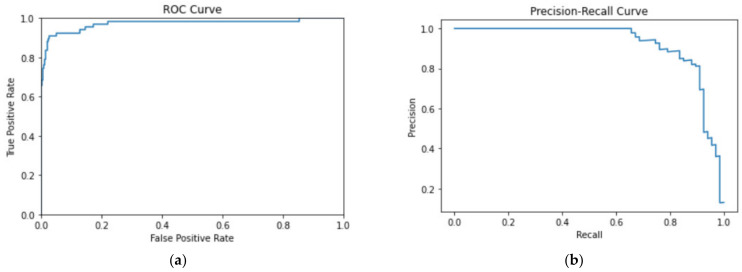
ROC curve performance of arrhythmia detection (**a**) ROC curve, and (**b**) PR curve.

Our model was evaluated with a test set containing a total of 592 samples. As seen in Table 2, the model outputs 59 true positives, 11 false positives, 514 true negatives, and 8 false negatives, with an accuracy of 96.79%, and precision and recall of 84.29% and 88.06%, respectively, resulting in an F1 score and MCC of 86.13% and 84.34%, as summarized in Table 3. Figure 7 shows the PR curve and the ROC curve with its AUC computed as 95.34%. Due to class imbalance, PR curve can be a better visualization of model performance than the ROC curve [26], so we present both plots in Figure 7 and verify that the PR curve, on top of the ROC curve, also shows a good model performance, despite class imbalance. The area under the PR curve (AUC-PR) is computed as 92.21%.

**Figure 8:**
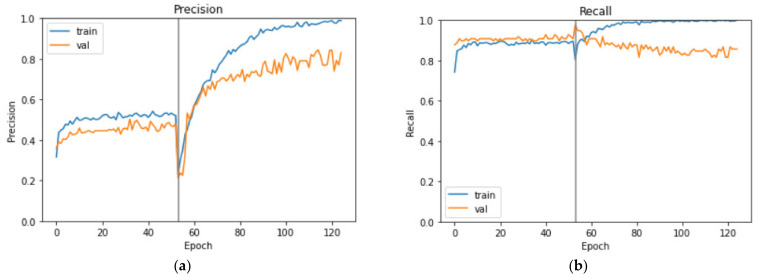
Evaluation metrics over each learning epoch (**a**) precision; (**b**) recall.

The precision and recall metrics over each learning epoch between the training and validation sets are shown in Figure 8. At the end of the training, the training set has a precision of 98.86%, a recall of 100.00%, and an *MCC* of 98.53%; the validation set has a precision of 83.17% a recall of 85.71%, and an *MCC* of 79.10%. From Figure 8, we observe that the transfer learning stage has a fairly high recall and a much lower precision, and the fine-tuning process is significantly improving the precision at a slight expense of recall for the validation set. The validation recall curve fluctuates little after epoch number 60 and is maintained above 80%. On the other hand, as the training precision curve approaches perfection, the validation precision curve maintains on the rise with its dips growing higher. Therefore, we experimented with a larger EfficientNet model, B1, but the performance metrics are inferior to those obtained from our B0 model. Table 3 also lists the model performance on the augmented dataset, and except for a slightly higher precision, AUC and AUC-PR, other metrics are inferior to those of the B0 model. Considering the extra computational time and resources on a dataset double the size for no significant improvement on model performance, we chose to only use the first set of spectrograms.

**Figure 9:**
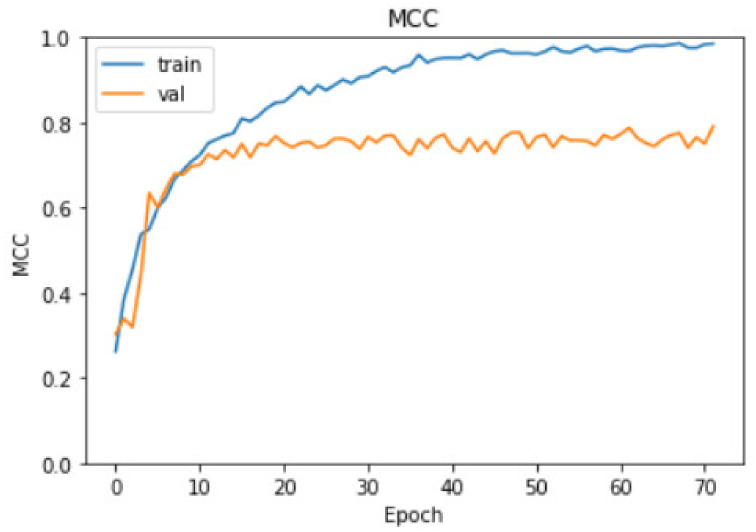
Matthews correlation coefficient curves.

For our B0 model, the *MCC* value over each epoch in the fine-tuning stage for both training and validation sets are plotted in Figure 9. We can observe that both scores are increasing throughout the iterations and the early stopping criteria on *MCC* gives us the maximum validation *MCC* (79.10%) with the training *MCC* at 98.53%, as indicated above.

### 3.3. Comparison of the Training Methods

We experimented with several training methods that included (i) transfer learning and fine-tuning (TL-FT), (ii) transfer learning as weights initialization (TL-WI), and (iii) random weights initialization (RWI). The results of these evaluations are summarized in Table 4. Compared to TL-FT, TL-WI correctly classified four more negative samples and four less positive samples. As the positive class is more important under class imbalance, TL-FT is more favorable and, additionally, it achieved slightly higher F1 score and MCC measure, although with a slightly lower AUC and AUC-PR. This further demonstrates the power of the transfer learning and fine-tuning approach that realized a better classification performance. In contrast, the RWI method fell short in terms of most of the performance metrics compared to TL-FT and TL-WI methods. This experiment also demonstrated that AUC can be misleading under class imbalance. Although the highest AUC is achieved using RWI method, the overall performance is inferior; rather, the AUC-PR is evidently a more reliable performance metric.

**Figure 10:**
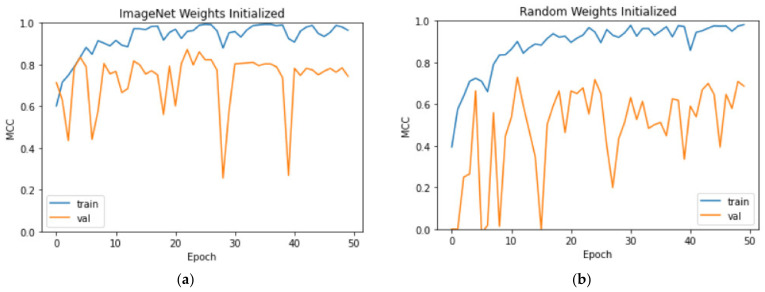
MCC curves with (**a**) TL-WI, and (**b**) RWI.

Figure 10 displays the MCC curves for both training and validation sets in experiments (ii) TL-WI and (iii) RWI. The number of training epochs is enough as MCC converges to 1, the maximum value. Compared to Figure 9, we observe that the validation MCC curve is much more stable with TL-FT, and in contrast, both the MCC curves with TL-WI and RWI exhibit fluctuations. This evidently demonstrates the robustness of the TL-FT approach and its advantage over directly training the models in experiments (ii) and (iii).

### 3.4. Examples of Incorrect Predictions

**Figure 11:**
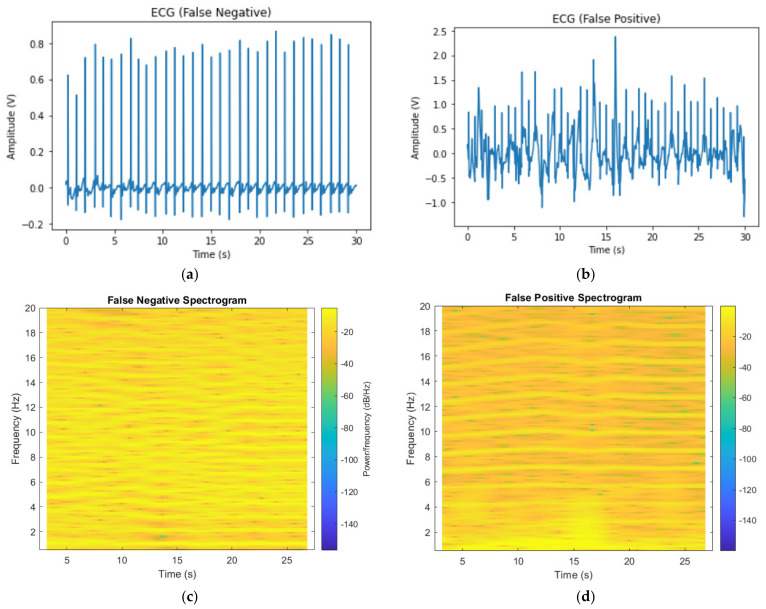
Examples of incorrect predictions: (**a**) False-negative ECG signal; (**b**) false-positive ECG signal; (**c**) false-negative spectrogram; (**d**) false-positive spectrogram.

Examples of a false negative and a false positive are shown in Figure 11. The left column is for an A-fib sample that was incorrectly predicted as normal by our model; the right column represents a normal sample incorrectly predicted as the A-fib class. For the raw ECG signal in Figure 11a, we observe high peaks and rather consistent heart rhythm. Therefore, the spectrogram was fairly smooth across the board and failed to result in a positive prediction. On the other hand, the right column contains the raw ECG signal in Figure 11b and the spectrogram of a normal sample falsely classified as A-fib (Figure 11d), where there is a noticeable irregularity around 15 s of the raw signal, shown as a lighter area in the spectrogram with a higher contrast compared to the other areas. This may have caused the incorrect model prediction.

### 3.5. Dropout Rate Changes

For the last dropout layer in our model, we experimented with different dropout rates and report the model performance in Table 5. A dropout rate of 0.8 returned the best performance in all metrics. A dropout rate of 0 is equivalent to not having the dropout layer and increasing the dropout rate from 0 continuously returned slightly more favorable model performance up until a dropout rate of 0.8. Going beyond 0.8 loses the ability to generalize and drops the model performance, as the performance metrics turned out lower when the dropout rate was 0.95.

### 3.6. Result Comparison with Related Works

For comparison, Table 6 summarizes how some related previous works in the literature using CNN or LSTM models addressed the issues of variable input length and the dataset imbalance. Many works attempted to fix the signal length, including Fan et al., who implemented padding and cropping to different signal lengths, which may have resulted in cleaner signals and higher performance metrics, as summarized in Table 7. Our work used raw signals as the input that included noise and variable lengths. The four-class classification results listed in Table 7 are individual F1 scores for the first three classes: normal, A-fib, and other, and the binary classification results included precision and recall.

## 4. Conclusions

The spectrogram-based alternative representation of ECG data was used in the A-fib detection. Such representation overcame the challenge of strong noise that existed in the raw ECG signals that led to our failed first attempt of training an RNN model. Utilizing the pretrained model with transfer learning and fine-tuning improved the performance and made the training process more robust, as shown by a comparison of results between different training methods. With high precision and recall metrics, our model produced effective classification results and potentially may assist doctors with the diagnosis of A-fib or provide a second opinion to the doctor’s judgement. The evaluation using MCC metrics and the PR curve further verified our model’s high performance under major class imbalance. Our model achieved excellent performance and demonstrated the ability to produce reliable predictions. As both the training and validation curves for precision continued to increase at the fine-tuning stage, this may suggest that a larger dataset may potentially further improve the model performance. In the application aspect, the predictions are completed within only a few seconds from the first data processing step, bandpass filter, to the final model output step, which is faster than a doctor can achieve in making a diagnosis. By training our model on spectrogram data based on raw ECG samples with various time lengths, we ensure that the model could perform effectively on test samples of any length, increasing its applicability within clinical settings. This algorithm, designed as a binary classifier, is trained to classify A-fib from normal ECGs. The model needs to be re-trained, tested, and validated on relevant datasets for binary or multi-class classifications of other arrythmia such as atrial or ventricular premature beats.

## Figures and Tables

**Table 1:** Data Profile from PhysioNet Challenge 2017: Statistics of the sample lengths (in seconds) for each type.

Type	# of Samples	Mean	Median	Max	Min	SD
Normal	5154	31.9	30	61	9	10.0
A-fib	771	31.6	30	60	10	12.5

**Table 2:** Confusion matrix.

	Predicted	Positive	Negative
Actual			
**Positive**		59	8
**Negative**		11	514

**Table 3:** Model results.

	Our B0 Model	Our B0 Model with Data Augmentation
Accuracy	96.79%	95.86%
Precision	84.29%	84.94%
Recall	88.06%	85.45%
F1 Score	86.13%	85.19%
MCC	84.34%	82.79%
AUC	95.34%	96.94%
AUC-PR	92.21%	92.65%

**Table 4:** Performance of our model with different weights initialization strategies.

	(i) TL-FT	(ii) TL-WI	(iii) RWI
TP	59	55	58
FP	11	7	31
TN	514	518	494
FN	8	12	9
Accuracy	96.79%	96.79%	93.24%
Precision	84.29%	88.71%	65.17%
Recall	88.06%	82.09%	86.57%
F1 Score	86.13%	85.27%	74.36%
MCC	84.34%	83.55%	71.50%
AUC	95.34%	96.24%	96.64%
AUC-PR	92.21%	92.42%	85.45%

**Table 5:** Model performance for different dropout rates.

Dropout Rate	Accuracy	Precision	Recall	F1 Score	MCC
0.95	96.62%	86.15%	83.58%	84.85%	82.96%
0.9	97.13%	85.71%	89.55%	87.59%	85.99%
0.8	97.30%	86.96%	89.55%	88.24%	86.72%
0.7	96.79%	83.33%	89.55%	86.33%	84.59%
0.5	96.79%	84.29%	88.06%	86.13%	84.34%
0	96.96%	90.16%	82.09%	85.94%	84.35%

**Table 6:** Key features of our model in comparison with related works in the literature.

Works	Model Type	Data Input	Addressed Data Imbalance?
Plesinger et al. [18]	CNN, NN, and BT	Raw signal	No
Kamaleswaran et al. [19]	13-layer 1D CNN	Repeating segments or zero-padding until 18,286 samples	No
Andreotti et al. [20]	ResNet	Truncated to the first minute	Yes
Fan et al. [21]	Multiscaled Fusion of CNN	Padded or cropped to fixed lengths	Yes
Xiong et al. [22]	16-layer 1D CNN	5-sec segments	No
Maknickas et al. [23]	LSTM	Divided into 46-timestep segments; padded shorter ones	No
Our work	EfficientNet B0	Raw signal	Yes

**Table 7:** Performance of our model in comparison with existing classifiers (- denotes not reported).

Works	Classification Type	Precision, Recall	F1 Scores *	F1 Score (Avg.)	MCC
Ref. [18]	4-class	-	91%, 80%, 74%	81%	-
Ref. [19]	4-class	-	91%, 82%, 75%	83%	-
Ref. [20]	4-class	-	93%, 78%, 78%	83%	-
Ref. [21]	2-class	85.43, 92.41% (padded/cropped to 5 s) 91.78%, 93.77% (padded/cropped to 20 s)	-	88.78% 92.76%	-
Ref. [22]	4-class	-	90%, 82%, 75%	82%	-
Ref. [23]	4-class	-	90%, 75%, 69%	78%	-
Our work	2-class	86.96%, 89.55%	-	88.24%	86.72%

* F1 score for the first 3 classes (normal, A-fib, and other).

## Data Availability

The data presented in this study are available on request from the corresponding author.
